# Demystifying the Salt-Induced Li Loss: A Universal Procedure for the Electrolyte Design of Lithium-Metal Batteries

**DOI:** 10.1007/s40820-023-01205-3

**Published:** 2023-10-24

**Authors:** Zhenglu Zhu, Xiaohui Li, Xiaoqun Qi, Jie Ji, Yongsheng Ji, Ruining Jiang, Chaofan Liang, Dan Yang, Ze Yang, Long Qie, Yunhui Huang

**Affiliations:** 1 State Key Laboratory of Material Processing and Die and Mold Technology, School of Materials Science and Engineering, Huazhong University of Science and Technology, Wuhan, 430074 Hubei People’s Republic of China; 2 Institute of New Energy for Vehicles, School of Materials Science and Engineering, Tongji University, Shanghai, 201804 People’s Republic of China; 3 Institute of Nanoscience and Nanotechnology, School of Physical Science and Technology, Central China Normal University, Wuhan, 430079 People’s Republic of China

**Keywords:** Li loss, Universal guideline, Electrolyte design, Li reversibility

## Abstract

Lithium (Li) metal electrodes show significantly different reversibility in the electrolytes with different salts. However, the understanding on how the salts impact on the Li loss remains unclear. Herein, using the electrolytes with different salts (e.g., lithium hexafluorophosphate (LiPF_6_), lithium difluoro(oxalato)borate (LiDFOB), and lithium bis(fluorosulfonyl)amide (LiFSI)) as examples, we decouple the irreversible Li loss (SEI Li^+^ and “dead” Li) during cycling. It is found that the accumulation of both SEI Li^+^ and “dead” Li may be responsible to the irreversible Li loss for the Li metal in the electrolyte with LiPF_6_ salt. While for the electrolytes with LiDFOB and LiFSI salts, the accumulation of “dead” Li predominates the Li loss. We also demonstrate that lithium nitrate and fluoroethylene carbonate additives could, respectively, function as the “dead” Li and SEI Li^+^ inhibitors. Inspired by the above understandings, we propose a universal procedure for the electrolyte design of Li metal batteries (LMBs): (i) decouple and find the main reason for the irreversible Li loss; (ii) add the corresponding electrolyte additive. With such a Li-loss-targeted strategy, the Li reversibility was significantly enhanced in the electrolytes with 1,2-dimethoxyethane, triethyl phosphate, and tetrahydrofuran solvents. Our strategy may broaden the scope of electrolyte design toward practical LMBs.

**Supplementary Information:**

The online version contains supplementary material available at 10.1007/s40820-023-01205-3.

## Highlights

- The loss mechanisms of irreversible Li in electrolytes with various salts (e.g., lithium hexafluorophosphate (LiPF_6_), lithium difluoro(oxalato)borate (LiDFOB), and lithium bis(fluorosulfonyl)amide (LiFSI)) are systemically revealed.
- A universal procedure for the electrolyte design of Li metal batteries is proposed: (i) decouple and find the main reason for the irreversible Li loss; (ii) add the corresponding electrolyte additive.

**Supplementary Information:**

The online version contains supplementary material available at 10.1007/s40820-023-01205-3.

## Graphical abstract

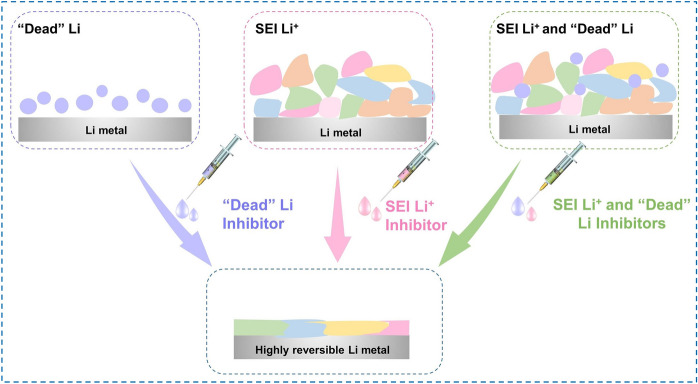

## Introduction

The need for batteries with higher energy density rekindles the research on lithium (Li) metal batteries (LMBs). However, the rapid formation and accumulation of irreversible Li loss during cycling lead to the deteriorating lifespan of LMBs, hindering their practical applications [1–5]. Fundamentally, the irreversible Li loss may attribute to two aspects: (i) the Li^+^-contained compounds (SEI Li^+^), including lithium fluoride (LiF), lithium oxide (Li_2_O), lithium alkyl carbonates, produced by the side reactions between Li metal and electrolyte [6, 7]; (ii) the electron-isolated “dead” Li, which is the product of the broken Li dendrites [8–10]. As yet, a range of electrolyte formulas have been developed to eliminate Li loss, with which the reversibility of Li metal electrodes is greatly improved with the coulombic efficiency (CE) increased from ~ 85% in carbonate electrolytes to > 99.5% in the ether, siloxane, and liquefied gas electrolytes [11–15]. However, the vague understanding on how the electrolyte components influence the Li reversibility slows down the further advance of the electrolytes.

As the main component of electrolyte, it is known the solvents with higher Gutmann donor number could be easily involved into the Li^+^ solvation structure and thus, affect the Li reversibility [16–18]. The solvent-induced Li loss has been previously investigated. Zhang and co-workers explored the differences in solvents (carbonate, sulfone, phosphate, and ether)-induced Li loss and found that the Li^+^ solvation structure is responsible for the different Li loss [19]. Using tetrahydrofuran (THF), dimethoxyethane (DME), 1,4-dioxane, 2-methyl-tetrahydrofuran as solvents, Huang and co-workers revealed the solvents with low polarity may alleviate the Li loss [3]. The pioneer works on solvents provide guidelines to select the proper solvents for LMBs [20–23]. On the other side, even with the optimized solvents, the use of different Li salts may still lead to jagged Li reversibility [24–27], which means there is still room to further optimize the salts for higher CE. The reported works concentrate more on how the salt initially contributes to the solid electrolyte interface [25, 26]. However, the discrepancy reasons in Li reversibility caused by salts during sequential cycles remain little understood. In this context, a deep understanding on how the salts affect the Li reversibility and the evolution of the “inactive” Li during the electrochemical processes become the key to taking the reversibility of Li metal to a higher level.

Herein, using electrolytes with lithium hexafluorophosphate (LiPF_6_), lithium difluoro(oxalato)borate (LiDFOB), or lithium bis(fluorosulfonyl)amide (LiFSI) salts as examples, we decoupled the evolution of the “inactive” Li during the electrochemical processes and unfolded the reasons for their differences. The accumulation of both SEI Li^+^ and “dead” Li in the porous interface of Li metal may be responsible to the irreversible Li loss for the Li metal in the electrolyte with LiPF_6_ salt. While for the electrolytes with LiDFOB and LiFSI salts, the accumulation of “dead” Li predominates the Li loss due to the formation consecutive and thin interface. Meanwhile, we also found lithium nitrate (LiNO_3_) and fluoroethylene carbonate (FEC) additives could, respectively, function as the “dead” Li and SEI Li^+^ inhibitors to pertinently restrain Li loss. Based on the above understanding, a Li-loss-targeted strategy was proposed, with which LMBs using electrolytes with DME, triethyl phosphate (TEP), and THF solvents achieved boosted reversibility.

## Experimental Section

### Electrolyte Preparation

The LiPF_6_ (DodoChem), LiDFOB (DodoChem), and LiFSI (DodoChem) electrolytes were prepared, respectively, by dissolving LiPF_6_, LiDFOB, or LiFSI in the mixture solvents of 1,2-dimethoxyethane (DME, DodoChem) and 1,1,2,2-tetrafluoroethyl-2,2,3,3-tetrafluoropropyl ether (TTE, DodoChem) with a volume ratio of 1:3.6. The molar ratios of LiPF_6_, LiDFOB, and LiFSI to DME are, respectively, 0.62, 0.45, and 0.93. The LiPF_6_–FEC electrolyte was prepared by adding 2 wt% fluoroethylene carbonate (FEC, DodoChem) into the LiPF_6_ electrolyte. The LiPF_6_–FEC–LiNO_3_ electrolyte was prepared by adding 2 wt% FEC and 0.2 M (mol L^−1^) lithium nitrate (LiNO_3_, DodoChem) into the LiPF_6_ electrolyte. The triethyl phosphate (TEP, DodoChem) electrolyte was prepared by dissolving 1 M LiFSI into TEP solvent. The TEP–FEC–LiNO_3_ electrolyte was prepared by dissolving 9 wt% FEC and 5 wt% LiNO_3_ into the TEP electrolyte. The THF electrolyte was prepared by dissolving 1 M LiFSI into THF solvent. The THF–LiNO_3_ electrolyte was prepared by dissolving 5 wt% LiNO_3_ into the THF electrolyte.

### Characterization Tests

The morphologies of the cycled Cu electrode collected from Li∥Cu cells were recorded by a field emission scanning electron microscope (SEM, Quanta 650 FEG). The composition and structure of the deposited Li layer were determined by the X-ray photoelectron spectrometer (XPS, AXIS-ULTRA DLD-600W) from a Cu electrode in Li∥Cu cells at 1 mA cm^−2^, 2 mAh cm^−2^. For titration gas chromatography measurement, the Li∥Cu cells after the 1st, 3rd, 5th, 7th, and 10th cycles were used to determine the evolution of Li loss following the steps: First, the Cu electrodes and separators disassembled from the Li∥Cu cells after different cycles were transferred into glass bottles with rubber stoppers and sealed with stainless steel rings in a glove box (the water and oxygen content below 0.1 ppm). Second, 0.5 mL ethanol and water mixture (volume ratio 1:2) was injected into each glass bottle to react with inactive Li before the test. Third, the gas (including H_2_) generated by the reaction is injected into the gas chromatograph (GC) (Shimadzu, GC-2014) through a gas-tight syringe, and the amount of injected gas was 1 mL. In order to ensure the accuracy of the test, each sample was tested three times, and the two similar H_2_ areas were regarded as the real data. Finally, the H_2_ area is converted to the mass of “dead” Li in inactive Li by the established calibration curve (Fig. S7). The SEI Li^+^ amount is calculated with Eq. (1):

1 $${\text{SEI}} \,{\text{Li}}^{ + } = {\text{Total}}\,{\text{inactive}}\, {\text{Li}} - ^{\prime\prime}{\text{dead}} \,{\text{Li}}^{\prime\prime}$$

### Electrochemical Measurements

Electrochemical cycling tests were carried out using CR2032 coin cells assembled in the glove box with H_2_O and O_2_ content below 0.1 ppm. All cells were measured on the Neware battery test system (CT-4008T, Shenzhen, China). Li∥Cu cells were used to investigate the Li plating/stripping reversibility by the constant-current protocol [28]. The CE is determined by the following steps. Specifically, 5 mAh cm^−2^ of Li was first deposited on Cu with a current density of 0.5 mA cm^−2^ and stripped to 1 V before depositing *Q*_*T*_ (5 mAh cm^−2^) of Li onto the Cu as a Li reservoir. Then, galvanostatic plating/stripping was carried out with a fixed areal capacity of *Q*_*C*_ (1 or 3 mAh cm^−2^) and a fixed current density (0.5 or 1 mA cm^−2^) for *n* (10) cycles, and finally stripping the final Li (*Q*_*S*_) to a cut-off voltage of 1 V. The CE over *n* cycles is calculated with Eq. (2):

2 $$CE = \frac{{Q_{S} + nQ_{C} }}{{Q_{T} + nQ_{C} }}$$

For Li∥LiNi_0.8_Co_0.1_Mn_0.1_O_2_ (NCM811) cells, NCM811 power was purchased from Shanshan New Energy Co., Ltd. The NCM811 cathodes with an areal mass loading of 6.4 mg cm^−2^ were prepared by blade-coating the slurry of mixing NCM811 (96 wt%), conductive carbon (2 wt%, DodoChem), and Poly(vinylidene fluoride) (PVDF, 2 wt% Arkema) binder in *N*-Methyl-2-pyrrolidone (NMP, Adamas reagent, ltd.) on carbon-coated Al current collectors (16 μm in thickness, Hefei Kejing Material Technology Co., Ltd.). The prepared NCM811 electrode was cut into discs (12 mm in diameter) and dried at 85 °C under vacuum overnight before use. For the measurement of Li∥NCM811 cells, the first two formation cycles at C/10 were conducted at 2.8 to 4.4 V, and then, the cells were charged to 4.4 V at C/3 and held at 4.4 V until the anodic current dropped below C/20 before discharged to 2.8 V at C/3 (1C = 200 mA g^−1^). CV curves for Li∥Cu cells were measured at a voltage range of − 0.3 to 1 V with a scan rate of 2 mV s^−1^. The electrochemical impedance spectroscopy (EIS) was measured on an electrochemical workstation (VMP3, BioLogic) with a frequency of 10^−1^ to 10^5^ Hz.

## Results and Discussion

### Electrochemical Characterizations of Li∥Cu and Li∥NCM811 Cells

In order to evaluate the difference in salt-induced Li loss, three Li salts, including LiPF_6_, LiDFOB, and LiFSI, are selected as examples. DME with low reduction voltage and superior Li compatibility, which can weaken the impact of solvents on our analysis results, is selected as the solvated solvent [29, 30]. TTE with a negligible solvability to Li salt is selected as diluent solvent, and the volume ratio of the DME to TTE is 1: 3.6. For the fair comparison of the influence of Li salts, the salt concentrations of all the electrolytes used in this work are 2 wt% less than the saturated ones, and the three electrolytes exhibit similar ion conductivity (Table S1).

**Fig. 1:**
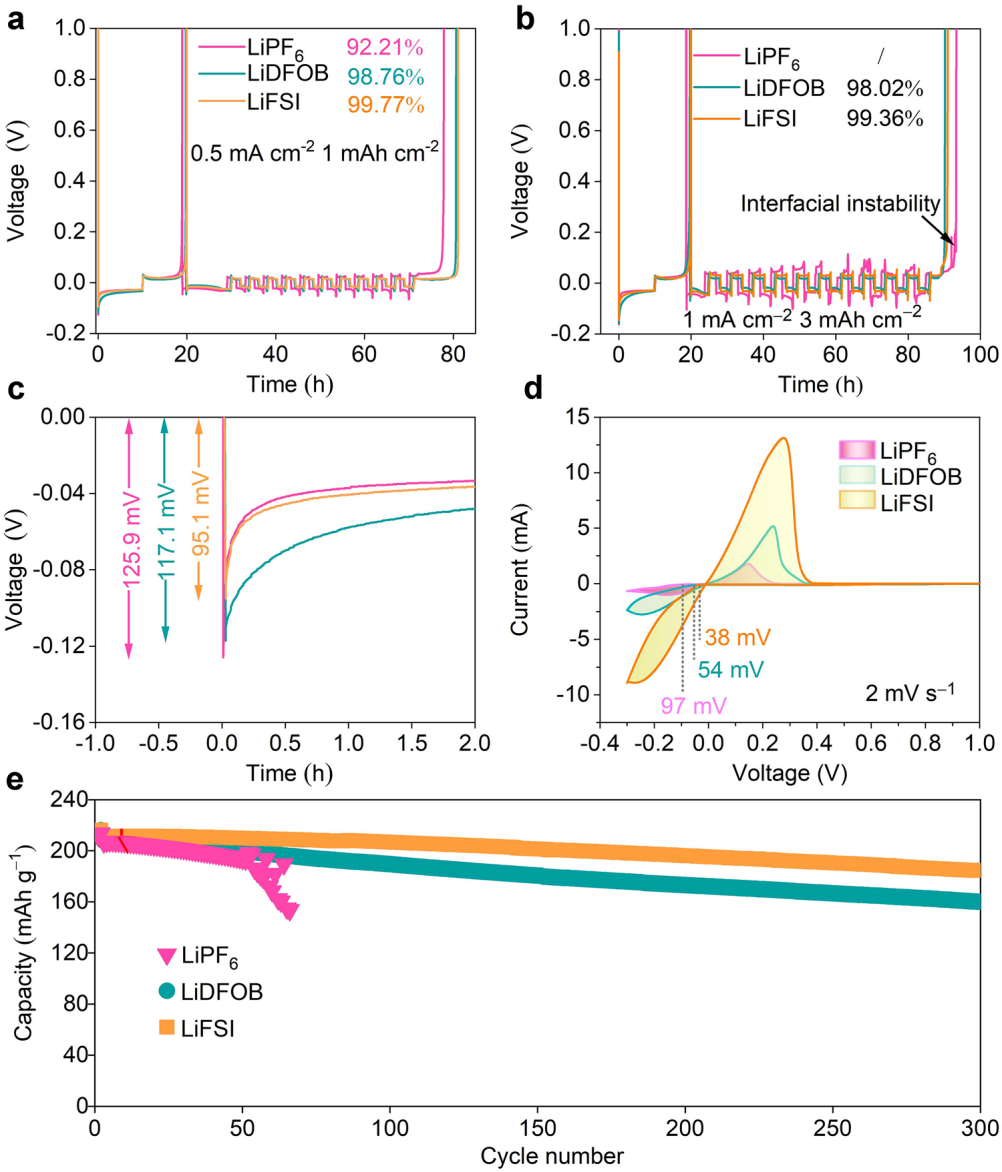
The electrochemical characterizations of Li∥Cu and Li∥NCM811 cells using LiPF_6_, LiDFOB, and LiFSI electrolytes. The average CE measured in Li∥Cu cells using LiPF_6_, LiDFOB, and LiFSI electrolytes with **a** 0.5 mA cm^−2^, 1 mAh cm^−2^, and **b** 1 mA cm^−2^, 3 mAh cm^−2^. **c** Li nucleation potential in LiPF_6_, LiDFOB, and LiFSI electrolytes. **d** Cyclic voltammetry (CV) measured by Li∥Cu cells using LiPF_6_, LiDFOB, and LiFSI electrolytes in a voltage range of − 0.3 and 1 V at 2 mV s^−1^. **e** The long-term cycling performance of Li∥NCM811 cells using LiPF_6_, LiDFOB, and LiFSI electrolytes at 0.3C after two formation cycles at 0.1C (1C = 200 mA g^−1^). (Color figure online)

Li∥Cu cells with a current density of 0.5 mA cm^−2^ and an areal capacity of 1 mAh cm^−2^ were first measured to evaluate Li reversibility in three electrolytes. As shown in Fig. 1a, Li∥Cu cells exhibit jagged reversibility from 92.21% to 99.77% in LiPF_6_, LiDFOB, and LiFSI electrolytes. The differences in Li reversibility are more prominent under a practical areal capacity (3 mAh cm^−2^) (Fig. 1b). Cell using LiPF_6_ electrolyte fails after 10 cycles, while cells in LiDFOB and LiFSI electrolytes still maintain relatively high CE (98.02% and 99.36%), which are consistent with the results of polarization voltages in Li∥Li cells (Fig. S1). These phenomena reveal salts do have a critical effect on Li plating/stripping behavior. The nucleation behavior of Li^+^ in different electrolytes was also studied, as shown in Fig. 1c. The nucleation overpotentials of Li plating in LiDFOB and LiFSI electrolytes (117.1 and 95.1 mV, respectively) are smaller than that (125.9 mV) in LiPF_6_ electrolyte, which displays a similar tendency with CV results of Li∥Cu cells (Fig. 1d). Such a result indicates that Li plating in LiPF_6_ electrolyte has a larger energy barrier than those in LiDFOB and LiFSI, thus leading to the generation of Li loss and inferior reversibility.

The salt-induced Li loss was also further monitored using Li∥ NCM811 full cells. The oxidation voltage of electrolyte is a prerequisite for high-voltage LMBs. As shown in Fig. S2, although the ether-based electrolyte is unstable above 4 V, the high ratio of the salt/solvent effectively suppresses the decomposition of ether solvents, which is consistent with previous reports [31–33]. The initial charge–discharge profiles of Li∥NCM811 cells are shown in Fig. S3. The initial discharging capacities in LiPF_6_, LiDFOB, and LiFSI electrolytes are 212.0, 214.3, and 213.3 mAh g^−1^, corresponding to initial CEs of 90.7%, 90.2%, and 87.1%, respectively. As the cycle proceeds to 300 cycles at 0.3C, the discrepancy in their discharging capacities is more distinct (Figs. 1e and S4). Li∥NCM811 cell using LiPF_6_ electrolyte shows a rapid decrease in capacity (74% after 66 cycles), which is worse than those using LiDFOB electrolyte (77% after 300 cycles) and LiFSI electrolyte (88% after 300 cycles).

Nyquist plots reveal partially variant reasons for electrochemical performance using three electrolytes. The impedance changes at the 5th, 30th, 80th, and 100th cycles are fitted by the equivalent circuit from Fig. S5, and the corresponding results are shown in Table S2. Cell in rapidly failing LiPF_6_ electrolyte exhibits a continuously increased ion diffusion impedance (Fig. S6), indicating that the products of side reactions are unremittingly generated on Li metal electrode. In contrast, stable ion diffusion impedance is found in LiDFOB and LiFSI electrolytes, which is responsible for less Li loss.

### Decoupling of Li Loss in LiPF_6_, LiDFOB, and LiFSI Electrolytes

**Fig. 2:**
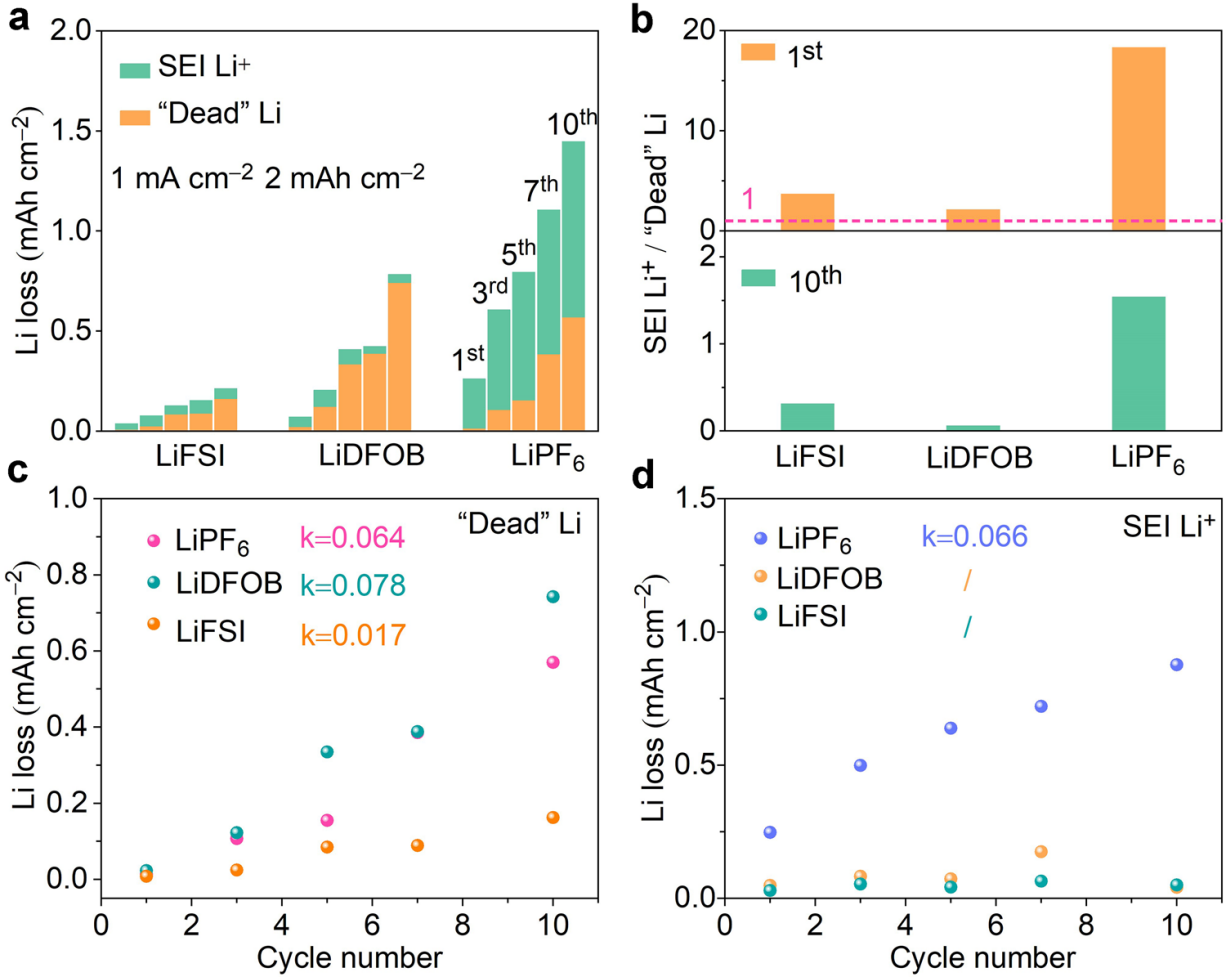
The decoupling of Li loss in LiPF_6_, LiDFOB, and LiFSI electolytes. **a** The evolution of SEI Li^+^ and “dead” Li in LiPF_6_, LiDFOB, and LiFSI electrolytes at the 1st, 3rd, 5th, 7th, and 10th cycles. **b** The ratios of the SEI Li^+^ to “dead” Li at the 1st and 10th cycles. **c** The “dead” Li as a function of cycle number in LiPF_6_, LiDFOB, and LiFSI electrolytes. **d** SEI Li^+^ as a function of cycle number in LiPF_6_, LiDFOB, and LiFSI electrolytes. (Color figure online)

Titration gas chromatograph (TGC) measurement was carried out to unveil the deeper discrepancy in salt-induced Li loss. Before the experiment, a highly linear calibration curve (*R*^2^ = 99.97%) is established to ensure the accuracy of measurement of “dead” Li (Fig. S7 and Table S3). As the results shown in Fig. 2a, the Li loss (including SEI Li^+^ and “dead” Li) in the LiPF_6_, LiDFOB, and LiFSI electrolytes increases continuously, which is consistent with the capacity decline during cycling. Figure 2b displays the detailed changes in Li loss caused by electrolytes with different salts. All cells exhibit larger Li loss from SEI Li^+^ than that from “dead” Li (SEI Li^+^ / “dead” Li > 1) in the first cycle, which is ascribed to the formation of solid electrolyte interphase (SEI), consistent with the reported conclusion in the literature [34]. Interestingly, with cycling proceeding to the 10th cycle, the evolution disparity in Li loss is more pronounced. The accumulation of “dead” Li dominates Li loss (SEI Li^+^ / “dead” Li < 1) in LiFSI and LiDFOB electrolytes. Nevertheless, Li loss in LiPF_6_ is a combined result of “dead” Li and SEI Li^+^ (SEI Li^+^ / “dead” Li = 1.5). The growth rates of “dead” Li and SEI Li^+^ are also quantified in Fig. 2c, d. The large growth rates of “dead” Li (0.064 mAh cm^−2^ per cycle) and SEI Li^+^ (0.066 mAh cm^−2^ per cycle) in LiPF_6_ electrolyte indicate the formation of unstable SEI, thus inducing serious side reactions between electrolyte and Li anode, which are responsible for rapid capacity decline during sequential cycles. In contrast, the slightly varied SEI Li^+^ demonstrates that dense interface can be formed in LiDFOB and LiFSI electrolytes. However, a larger growth rate of “dead” Li from LiDFOB (0.078 mAh cm^−2^ per cycle) than that from LiFSI electrolyte (0.017 mAh cm^−2^ per cycle) results in their difference in electrochemical performance.

### Li Deposition Morphologies and Characterization of SEI

**Fig. 3:**
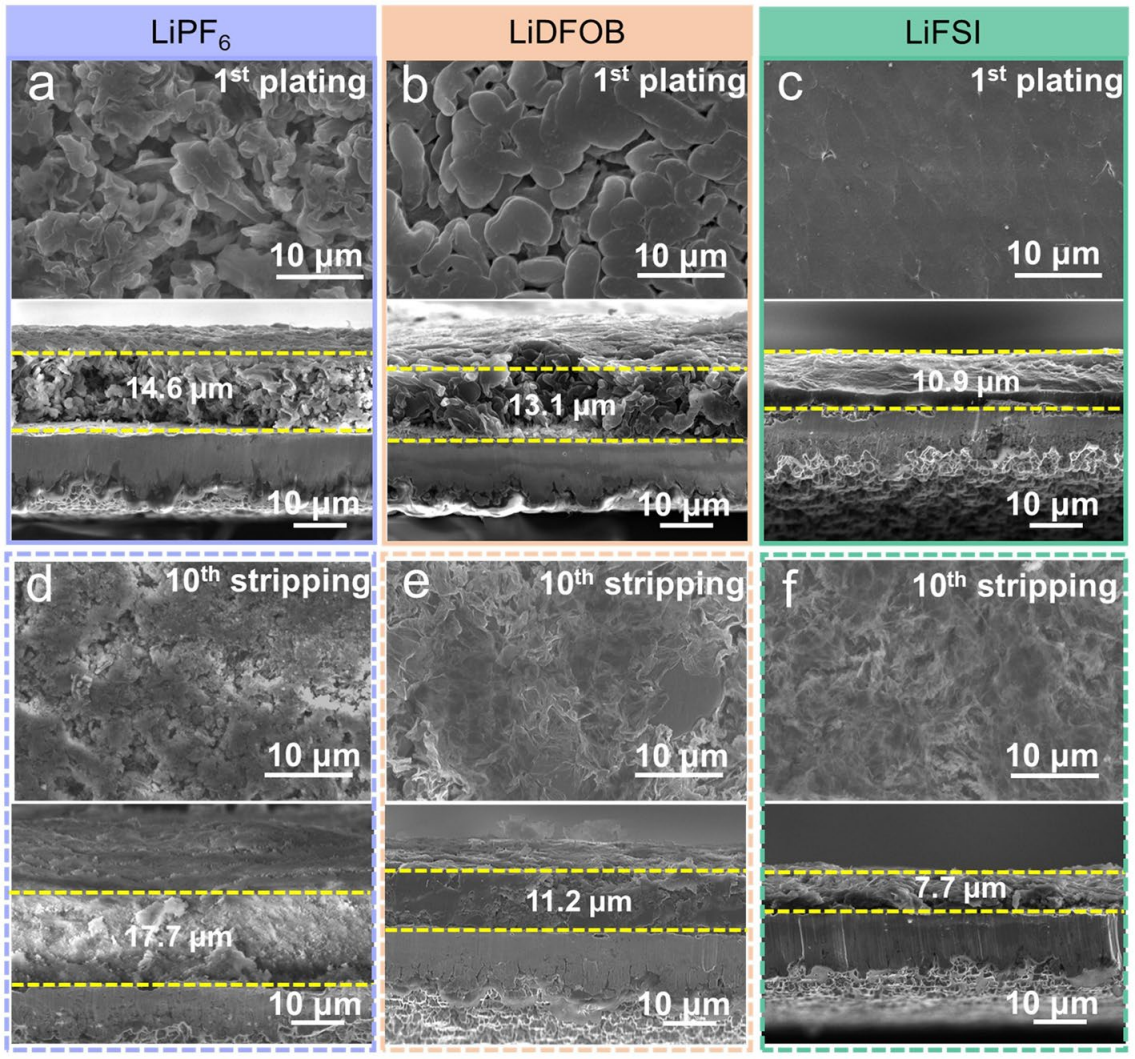
The morphological characterization of the 1st plating and 10th stripping Cu electrodes obtained from Li∥Cu cells at 1 mA cm^−2^ and 2 mAh cm^−2^. Surface and cross-section morphologies of the 1st plating Cu electrode and the 10th stripping Cu electrode in **a, d** LiPF_6_, **b, e** LiDFOB, and **c, f** LiFSI electrolytes

SEM was used to detect different evolution reasons for Li loss in electrolytes with LiPF_6_, LiDFOB, and LiFSI salts. As the morphology observed in the LiPF_6_ electrolyte (Fig. 3a), dendritic Li with various lengths randomly stacks together after the 1st plating. It is difficult to suppress the side reactions between the electrolyte and the plating Li, leading to a thick Li plating layer (~ 14.6 μm). When the plated Li is stripped to the counter electrode, a large amount of “dead” Li and SEI Li^+^ remains on the Cu substrate (Fig. 3d), and the thickness of the residues is 17.7 μm. These results indicate that the single LiPF_6_ salt is insufficient to form a stable interface. In LiDFOB electrolyte, these problems are significant changes. As shown in Fig. 3b, the 1st plating morphology of Li in LiDFOB electrolyte shows a stacked blocky morphology, which lessens the formation of Li dendrites, thus resulting in a 13.1 μm plating layer of Li. After the 10th stripping (Fig. 3e), consecutive and fewer residues (thickness: 11.2 μm) remain on the Cu substrate. In contrast, dense Li plating morphology at the 1st cycle (Fig. 3c) and smaller residues (vs. that in LiDFOB electrolyte) at the 10th cycle (Fig. 3f) endow the improved electrochemical performance of the cell using LiFSI electrolyte.

**Fig. 4:**
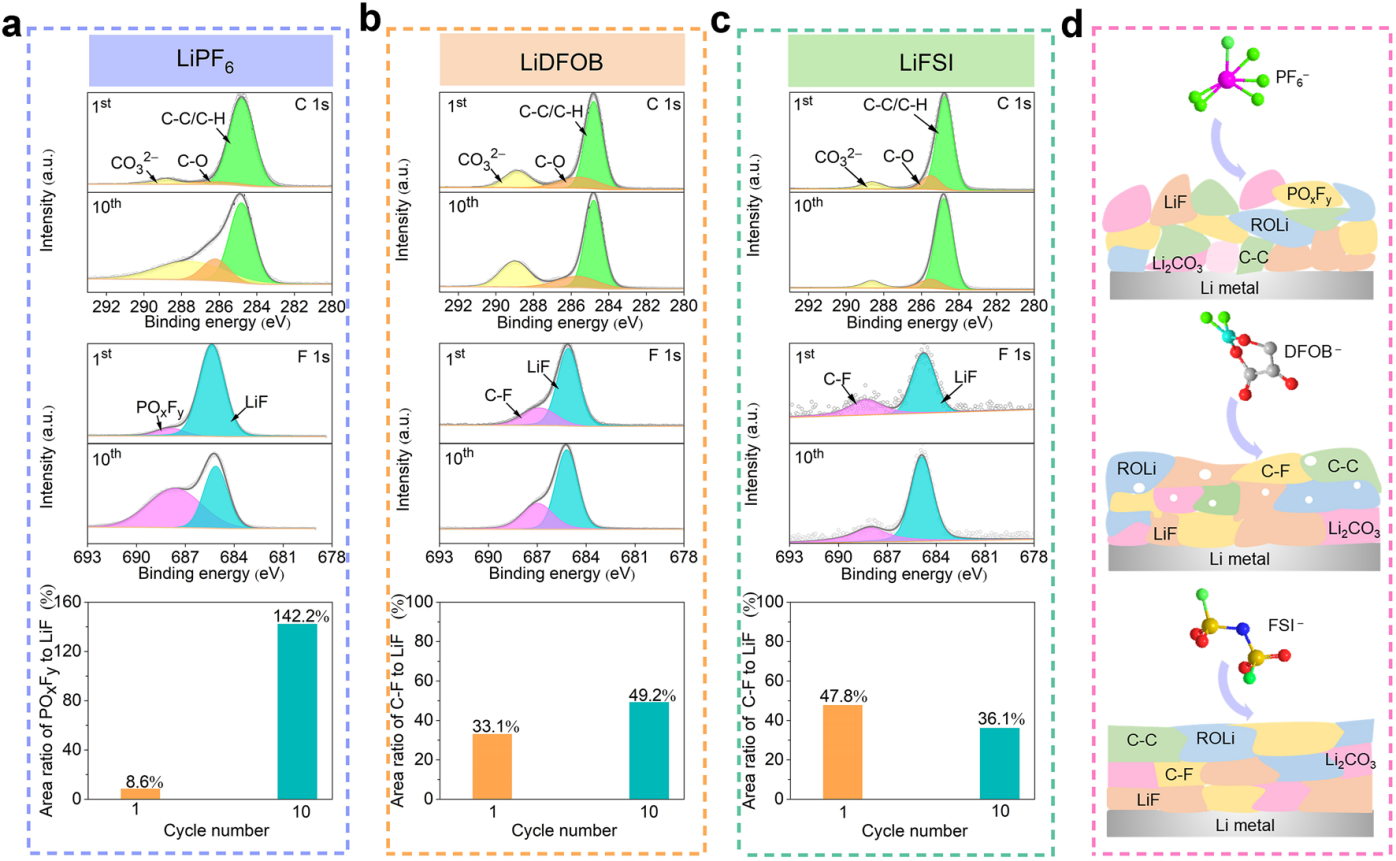
The characterization of interfacial components of cycled Cu electrodes. The C 1s and F 1s XPS spectra of the cycled Cu electrodes obtained from Li∥Cu cells using **a** LiPF_6_, **b** LiDFOB, and **c** LiFSI electrolytes, and the corresponding area ratios of the PO_*x*_F_*y*_ or C–F peak to LiF peaks after the 1st and 10th plating. **d** Schematic illustration of the interface formed in LiPF_6_, LiDFOB, and LiFSI electrolytes (The green, pink, dark red, gray, yellow, cyan, and blue represent F, P, O, C, S, B, and N atoms, respectively). (Color figure online)

The evolution of SEI Li^+^ and “dead” Li is also closely related to the changing interfacial components. Therefore, XPS is carried out on cycled Cu electrodes. As XPS of cycled Cu electrode in LiPF_6_ electrolyte displayed in Figs. 4a and S8, the peaks, including C–O at 286.2 eV and CO_3_^2−^ at 287.5 eV in C 1*s*, and C–O at 532.7 eV in O 1*s*, dramatically increase after the 10th plating (compared with those after the 1st plating), indicating the formed interface in LiPF_6_ electrolyte is unstable. The interfacial instability can also be explained by the increased PO_*x*_F_*y*_ peak and the large area ratio of the PO_*x*_F_*y*_ to LiF peak (142.2%) in F 1s after the 10th plating. The PO_*x*_F_*y*_ peak produced by the decomposition of PF_5_^−^ and PF_6_^−^, which is unavoidably leads to the formation of HF [22]. Its leaching effect may cause more voids on Li metal interface, thus resulting in the rapid growth of SEI Li^+^ and “dead” Li [24]. The interfacial behavior is markedly ameliorated in the LiDFOB electrolyte. As shown in Figs. 4b and S9, the peaks (C–O and CO_3_^2−^ peaks in C 1s spectra, and C–O peak in O 1s spectra) and the area ratio of the C–F to LiF peak after the 10th plating increase slightly (compared with those at the 1st plating), indicating stable interface can be formed in LiDFOB electrolyte to prevent the growth of SEI Li^+^, thus improving Li stability. The further improvement of interfacial stability in LiFSI electrolyte can be confirmed by negligible composition change on Li metal anode, which is responsible for the lowest growth rates of “dead” Li among the three electrolytes (Figs. 4c and S10). These results are consistent with TGC and SEM results.

The above results reveal the remarkable impact of salt on Li loss. The evolution differences of Li loss in the interface are also concluded by schematic illustration (Fig. 4d). The porous interface in LiPF_6_ electrolyte accelerates the evolution of SEI Li^+^ and “dead” Li, thus leading to premature failure of LMBs. In LiDFOB, consecutive and thin interfaces effectively suppress side reactions and prevent the dynamic evolution of SEI Li^+^. However, the mass “dead” Li deteriorates its electrochemical performance. The stable and compact interface in LiFSI electrolyte retards the evolution of SEI Li^+^ and slows down the growth rates of “dead” Li, greatly improving Li reversibility.

### Characterizations of Li loss and CE

**Fig. 5:**
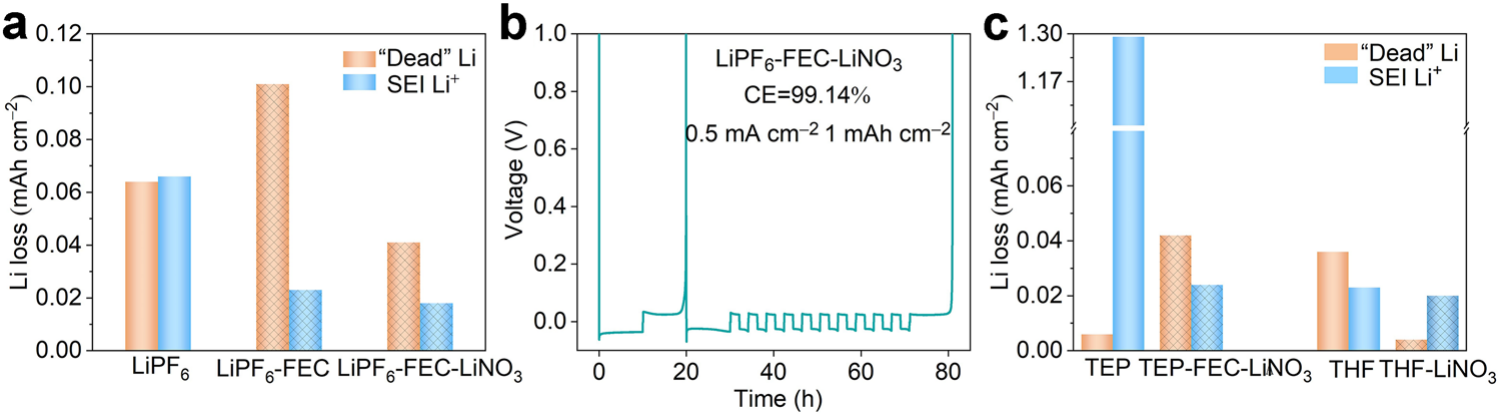
The characterizations of Li loss and CE. **a** The characterization of SEI Li^+^ and “dead” Li in the LiPF_6_, LiPF_6_–FEC, and LiPF_6_–FEC–LiNO_3_ electrolytes. **b** CE measured using Li∥Cu cell in LiPF_6_–FEC–LiNO_3_ electrolyte. **c** The characterization of SEI Li^+^ and “dead” Li in the TEP, TEP–FEC–LiNO_3_, THF, and THF–LiNO_3_ electrolytes. (Color figure online)

Based on the above discussions, we demonstrate that behaviors of the salt-induced Li loss during cycling vary with the salts, which also implies that we should pertinently select additives to eliminate Li loss. FEC and LiNO_3_ as excellent film-forming additives have been previously investigated [35–38]. As a proof of concept, we introduced them into LiPF_6_ electrolyte. Interestingly, a new phenomenon is observed that they could, respectively, function as the “dead” Li and SEI Li^+^ inhibitors to pertinently restrain Li loss. As shown in Figs. 5a and S11, the reduced SEI Li^+^ and increased “dead” Li (compared with the Li loss in the LiPF_6_ electrolyte) are found in the FEC-involved LiPF_6_ electrolyte (LiPF_6_–FEC electrolyte, the LiPF_6_ electrolyte with 2 wt% FEC), indicating FEC may be an inhibitor of SEI Li^+^. Interestingly, a significant reduction in “dead” Li can also be confirmed after further adding the LiNO_3_ into LiPF_6_–FEC electrolyte (LiPF_6_–FEC–LiNO_3_ electrolyte, the LiPF_6_ electrolyte with 2 wt% FEC and 0.2 M LiNO_3_), implying the LiNO_3_ may be an inhibitor of “dead” Li. Therefore, Li∥Cu cell, benefitting from the restrained Li loss, obtains a significantly improved with CE increased from 92.21% in LiPF_6_ electrolyte to 99.14% in LiPF_6_–FEC–LiNO_3_ electrolyte (Fig. 5b). These conclusions demonstrate that the Li loss can be eliminated by adding targeted additives.

The universality of the Li-loss-targeted strategy is further validated by TEP and THF electrolytes. As the results of TGC from the TEP electrolyte (1 M LiFSI in TEP) shown in Figs. 5c and S12, the infinite growth of SEI Li^+^, which is ascribed to poor compatibility between TEP and Li anode, appears in the TEP electrolyte, even hiding the information of “dead” Li. After the introduction of the inhibitors of LiNO_3_ and FEC (1 M LiFSI in TEP with 9 wt% FEC and 5 wt% LiNO_3_, TEP–FEC–LiNO_3_), the progressiveness of electrolyte is as expected. For THF electrolyte (1 M LiFSI in THF), the low reduction voltage of THF solvent promotes the formation of the stable interface on the Li metal anode. Consequently, TGC results show that the growth of “dead” Li is the dominant factor for the Li loss in THF electrolyte. For the growth of “dead” Li in the THF electrolyte, we realize the high Li reversibility by adding sole LiNO_3_ in the THF electrolyte (the THF electrolyte with 5 wt% LiNO_3_, THF–LiNO_3_ electrolyte). These results demonstrate that advanced electrolytes can be designed in light of the interfacial evolution of Li loss, which paves the way for developing advanced electrolytes to realize the practical applications of LMBs.

## Conclusions

In this work, we revealed the discrepancy of salt (LiPF_6_, LiDFOB, and LiFSI)-induced Li loss during cycling. It is found that the accumulation of “dead” Li and SEI Li^+^ in LiPF_6_ electrolyte deteriorates the overall cell performance. However, the evolution of “dead” Li in both LiDFOB and LiFSI electrolytes is responsible for the fading performance. By introducing sequentially FEC and LiNO_3_ into LiPF_6_ electrolyte, we successfully eliminate SEI Li^+^ or/and “dead” Li, ultimately achieving enhanced Li reversibility in LiPF_6_ electrolyte. Based on above understanding, a Li-loss-targeted strategy that is applying FEC and LiNO_3_ as inhibitors of SEI Li^+^ and “dead” Li, respectively, is proposed to purposefully eliminate Li loss, with which LMBs using electrolytes with DME, TEP, and THF solvents achieve boosted Li reversibility. This work provides new insight into the advanced electrolyte design for high-energy–density LMBs by demystifying Li loss.

### Supplementary Information

Below is the link to the electronic supplementary material.

Supplementary file1 (PDF 711 KB)
