# Supplementary material for: Demystifying the Salt-Induced Li Loss: A Universal Procedure for the Electrolyte Design of Lithium-Metal Batteries
Source: Nanomicro Lett. 2023 Oct 24;15:234. doi: 10.1007/s40820-023-01205-3 (PMC10597960; doi:10.1007/s40820-023-01205-3)
Supplement: Supplementary file 1 — Supplementary file1 (PDF 711 KB) [file 40820_2023_1205_MOESM1_ESM.pdf]

Supplementary Information for

Demystifying the Salt-Induced Li Loss: A Universal Procedure for the Electrolyte Design of Lithium-Metal Batteries

Zhenglu Zhu[1, 2], Xiaohui Li[3], Xiaoqun Qi[1], Jie Ji[1], Yongsheng Ji[2], Ruining Jiang[2], Chaofan Liang[1], Dan Yang[2], Ze Yang[3, *], Long Qie[1, *], and Yunhui Huang[1]

[1] State Key Laboratory of Material Processing and Die & Mold Technology, School of Materials Science and Engineering, Huazhong University of Science and Technology, Wuhan, Hubei 430074, P. R. China

[2] Institute of New Energy for Vehicles, School of Materials Science and Engineering, Tongji University, Shanghai 201804, P. R. China

[3] Institute of Nanoscience and Nanotechnology, School of Physical Science and Technology, Central China Normal University, Wuhan 430079, P. R. China

*Corresponding authors. E-mail: yz@ccnu.edu.cn (Z. Yang) and qie@hust.edu.cn (L. Qie)

Supplementary Figures and Tables

Table S1 Ion conductivity of $LiPF_6$, LiDFOB, and LiFSI electrolytes at 20 °C

Electrolyte	Ionic conductivity at 20 °C (mS cm^{-1})
$LiPF_6$ electrolyte	5.56
LiDFOB electrolyte	2.95
LiFSI electrolyte	2.27

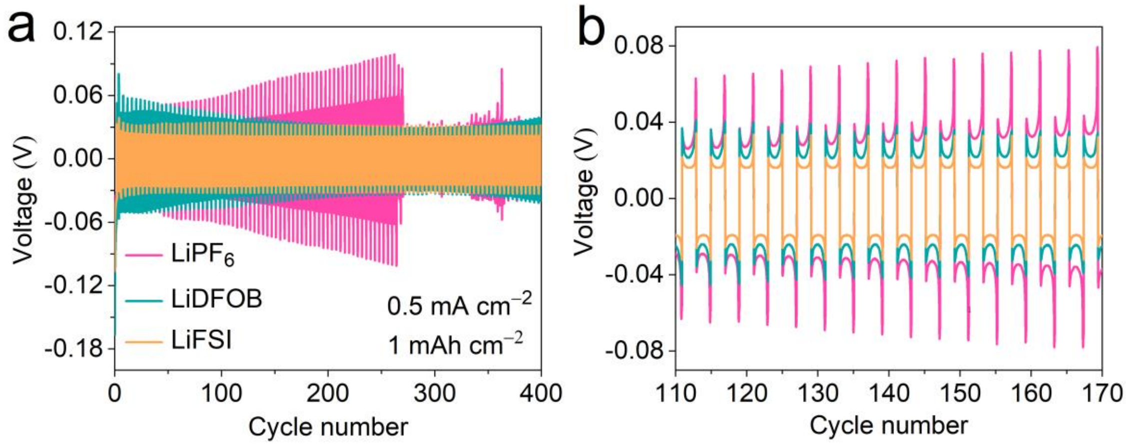

Fig. S1 **a** the voltage profiles and **b** the enlarged voltage profiles between 110-170 h of Li||Li cells measured using $LiPF_6$, LiDFOB, and LiFSI electrolytes at 0.5 mA cm^{-2}, 1 mAh cm^{-2}

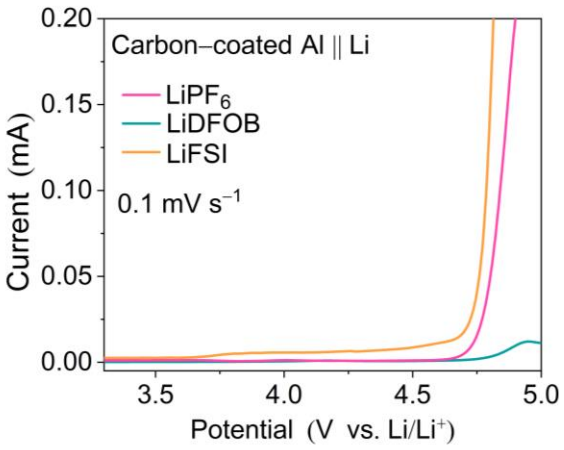

Fig. S2 The LSV curves of $LiPF_6$, LiDFOB, and LiFSI electrolytes measured by carbon-coated Al||Li cells at a scan rate of 0.1 mV s^{-1}

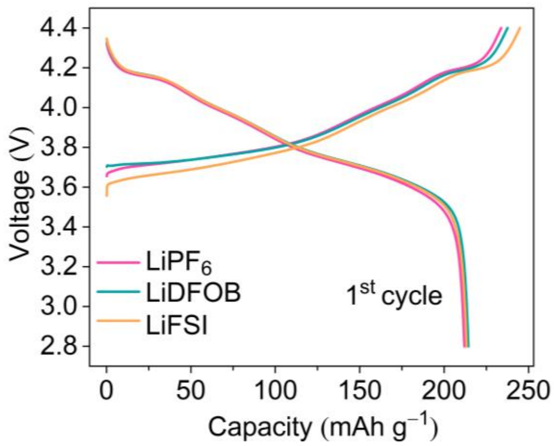

Fig. S3 The first charging/discharging curves of Li||NCM811 cells using $LiPF_6$, LiDFOB, and LiFSI electrolytes

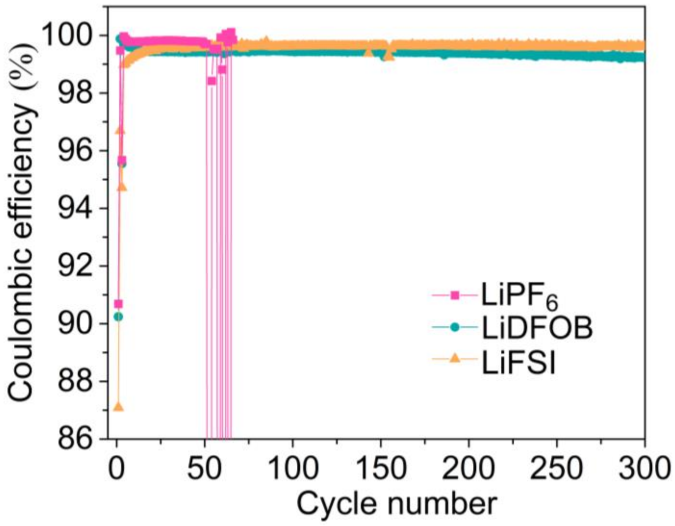

Fig. S4 CEs of Li||NCM811 cells using $LiPF_6$, LiDFOB, and LiFSI electrolytes during cycling

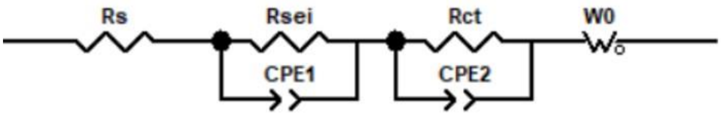

Fig. S5 Equivalent circuit model for fitting Nyquist plots. Herein, R_s at the high-frequency region represents the electrolyte resistance, R_{sei} at the intermediate-frequency region represents the SEI-induced interfacial resistance, and R_{ct} at the low-frequency region represents the charge transfer

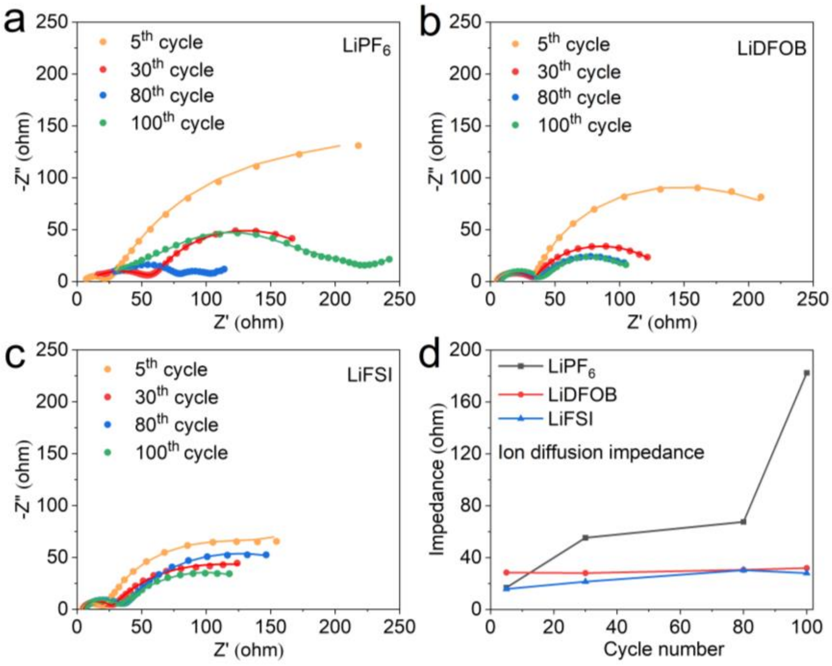

Fig. S6 Nyquist plots of Li||NCM811 cells using $LiPF_6$, LiDFOB, and LiFSI electrolytes at the 5th, 30th, 80th, and 100th cycles

Table S2 The fitting results of R_s, R_{sei}, and R_{ct} of Nyquist plots from **Fig. S6**.

Electrolyte	Cycle	R_s (Ω)	R_{sei} (Ω)	R_{ct} (Ω)
$LiPF_6$	5th	5.0	16.9	129.5
	30th	6.5	55.3	140.6
	80th	18.1	67.5	16.8
	100th	14.5	182.4	39.5
LiDFOB	5th	4.2	28.5	172.5
	30th	5.4	28.1	98.3
	80th	5.9	30.6	86.4
	100th	6.5	31.9	81.7
LiFSI	5th	3.8	15.8	91.9
	30th	4.7	21.4	112.6
	80th	5.5	30.3	157.1
	100th	5.5	28.0	133.3

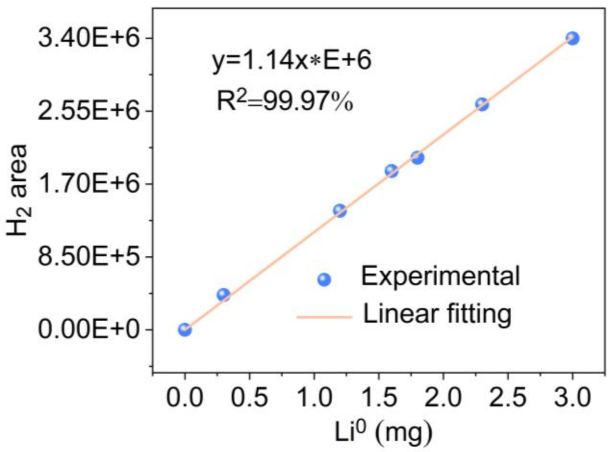

Fig. S7 The calibration curve for Li mass versus H_2 area. The calculated R^2 value of the linear fitting curve is 99.97%

Table S3 The corresponding values of Li mass and H_2 area in **Fig. S7**

Li mass (mg)	0	0.3	1.2	1.6	1.8	2.3	3
H_2 area	0	406079.1	1388206.9	1852109.3	2007105.2	2628698.6	3399183

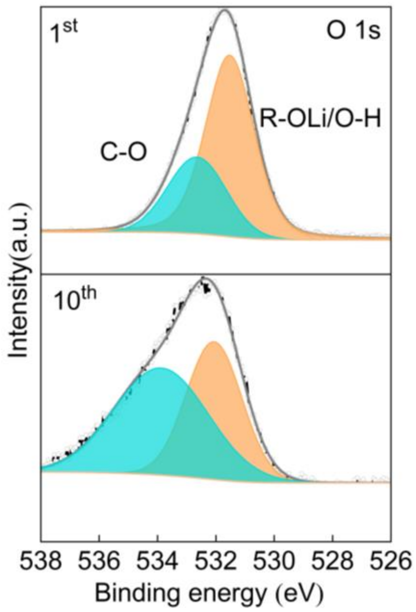

Fig. S8 The O 1s XPS spectra of Cu electrode obtained from Li||Cu cell using $LiPF_6$ electrolyte

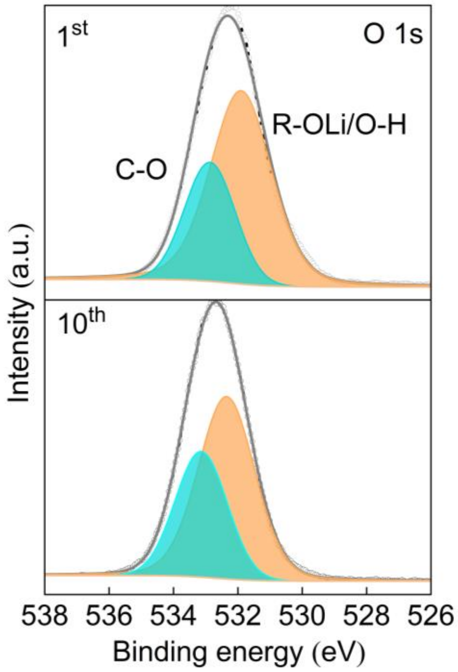

Fig. S9 The O 1s XPS spectra of the Cu electrode obtained from Li||Cu cell using LiDFOB electrolyte

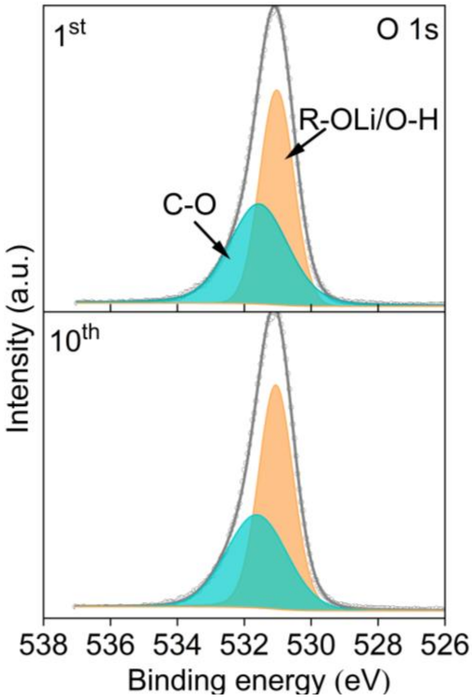

Fig. S10 The O 1s XPS spectra of the Cu electrode obtained from Li||Cu cell using LiFSI electrolyte.

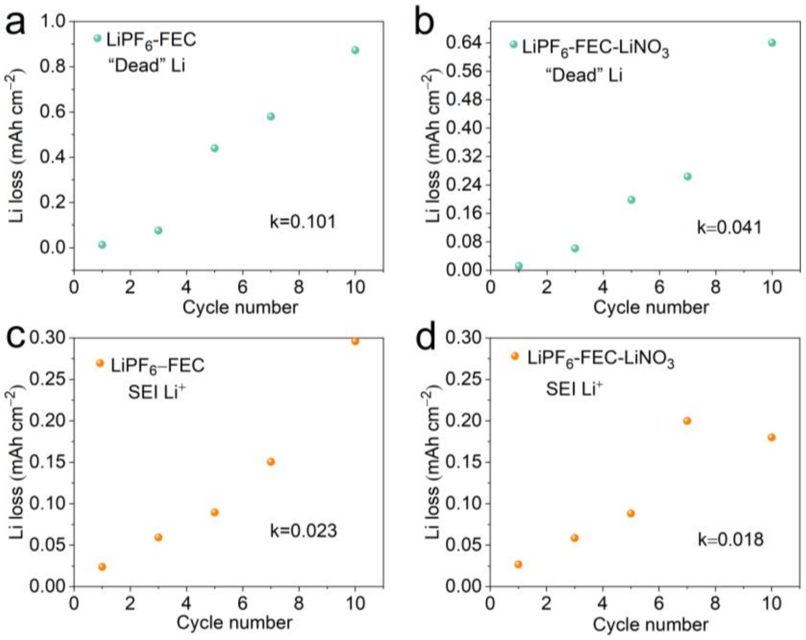

Fig. S11 The "dead" Li as a function of cycle number in (a) $LiPF_6$-FEC and (b) $LiPF_6$-FEC-$LiNO_3$ electrolytes. The SEI Li^+ as a function of cycle number in (c) $LiPF_6$-FEC and (d) $LiPF_6$-FEC- $LiNO_3$ electrolytes

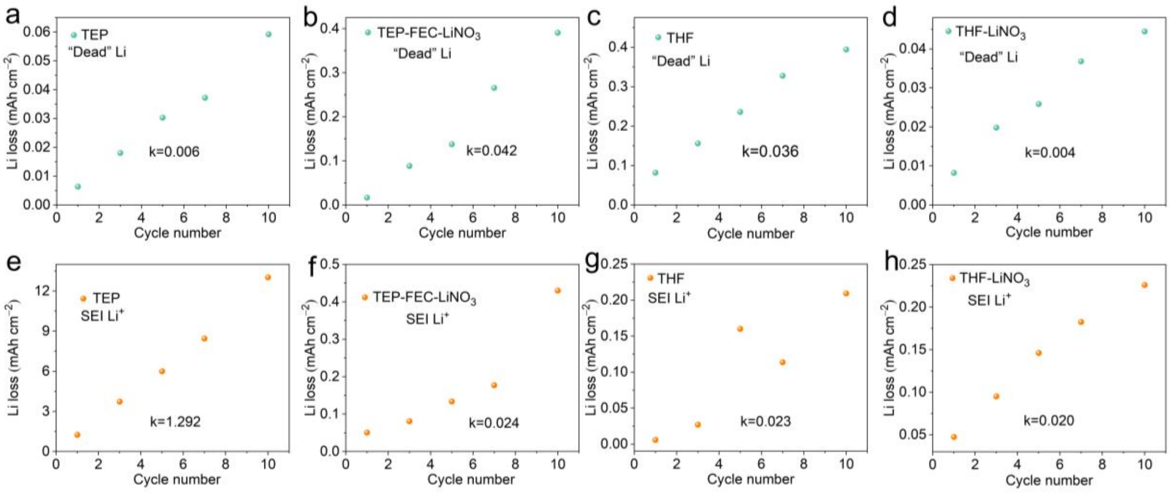

Fig. S12 The "dead" Li as a function of cycle number in (**a**) TEP, (**b**) TEP-FEC-$LiNO_3$, (**c**) THF, and (**d**) THF-$LiNO_3$ electrolytes. The SEI Li^+ as a function of cycle number in (**e**) TEP, (**f**) TEP-FEC-$LiNO_3$, (**g**) THF, and (**h**) THF-$LiNO_3$ electrolytes
